# Acupuncture to improve live birth rates for women undergoing *in vitro* fertilization: a protocol for a randomized controlled trial

**DOI:** 10.1186/1745-6215-13-60

**Published:** 2012-05-18

**Authors:** Caroline A Smith, Sheryl de Lacey, Michael Chapman, Julie Ratcliffe, Robert J Norman, Neil Johnson, Gavin Sacks, Jane Lyttleton, Clare Boothroyd

**Affiliations:** 1 Centre for Complementary Medicine Research, University of Western Sydney, Locked Bag, 1797, Penrith South, DC, NSW 2751, Australia; 2 School of Nursing & Midwifery, Flinders University, GPO Box 2100, Adelaide, South Australia, 5001, Australia; 3 School of Women’s and Children’s Health, University of New South Wales, St George Hospital, IVF Australia, Southern Sydney, South Street Kogarah, NSW 2217, Australia; 4 Flinders Clinical Effectiveness, Flinders University, Bedford Park, South Australia, 5001, Australia; 5 Robinson Institute, University of Adelaide, Norwich House, 55 King William Road, North Adelaide, South Australia, 5006, Australia; 6 Fertility Plus, Auckland District Health Board; Repromed Auckland, University of Auckland, Auckland, New Zealand; 7 School of Women’s and Children’s Health, University of New South Wales, St George Hospital, South Street, Kogarah, New South Wales, 2217, Australia; 8 Paddington Medical Centre, 198 Oxford St, Paddington, New South Wales, 2021, Australia; 9 Assisted Conception Australia, Greenslopes Private Hospital, Suite 9A, Administration Building, Brisbane, QLD, 4120, Australia

## Abstract

****Background**:**

IVF is a costly treatment option for women, their partners, and the public. Therefore new therapies that improve reproductive and health outcomes are highly desirable. There is a growing body of research evaluating the effect of acupuncture administered during IVF, and specifically on the day of embryo transfer (ET). Many trials are heterogeneous and results inconsistent. There remains insufficient evidence to determine if acupuncture can enhance live birth rates when used as an adjunct to IVF treatment.

The study will determine the clinical effectiveness of acupuncture with improving the proportion of women undergoing IVF having live births. Other objectives include: determination of the cost effectiveness of IVF with acupuncture; and examination of the personal and social context of acupuncture in IVF patients, and examining the reasons why the acupuncture may or may not have worked.

****Methods**:**

We will conduct a randomized controlled trial of acupuncture compared to placebo acupuncture.

Inclusion criteria include: women aged less than 43 years; undergoing a fresh IVF or ICSI cycle; and restricted to women with the potential for a lower live birth rate defined as two or more previous unsuccessful ETs; and unsuccessful clinical pregnancies of quality embryos deemed by the embryologist to have been suitable for freezing by standard criteria. Women will be randomized to acupuncture or placebo acupuncture. Treatment is administered on days 6 to 8 of the stimulated cycle and two treatments on the day of ET. A non-randomized cohort of women not using acupuncture will be recruited to the study. The primary study outcome is the proportion of women reporting a live birth. Secondary outcomes include the proportion of women reporting a clinical pregnancy miscarriage prior to 12 weeks, quality of life, and self-efficacy. The sample size of the study is 1,168 women, with the aim of detecting a 7% difference in live births between groups (*P* = 0.05, 80% power).

****Discussion**:**

There remains a need for further research to add significant new knowledge to defining the exact role of certain acupuncture protocols in the management of infertility requiring IVF from a clinical and cost-effectiveness perspective.

****Clinical Trial Registration**:**

Australian and New Zealand Clinical Trial Registry ACTRN12611000226909

## **Background**

There are a greater number of women now attempting pregnancy at an older age when they are less fertile; and consequently a growing number of couples accessing Assisted Reproductive Technologies (ART). In Australia during 2008, there were 61,929 ART cycles, however only 22.6% resulted in a clinical pregnancy and 17.2% resulted in live deliveries [1]. *In vitro* fertilization (IVF) is a resource-intensive and costly treatment option for both women and their families, and for public healthcare expenditure, and there is a significant personal cost to couples undergoing repeated IVF cycles. Therefore new therapies that improve reproductive and health outcomes are highly desirable.

Complementary therapies and medicines are used by individuals with infertility and eight surveys report prevalence rates between 13% and 82% [2-9]. Acupuncture has become an emerging therapy used as an adjunct to IVF. A survey of UK acupuncturists indicates 15% provide fertility support, and for some practitioners this has become a large proportion of their caseload [10]. Eighty percent of acupuncturists reported most of this work was related to assisted conception.

There is a growing body of research evaluating the effect of acupuncture administered during IVF, and specifically on the day of embryo transfer (ET). The first systematic review was published in 2008 and this review found that acupuncture as an adjunct to ET was associated with statistically and clinically significant increases in the pregnancy, ongoing pregnancy, and live birth rates [11]. Although these findings were encouraging they were somewhat preliminary, and highlighted a need for further placebo controlled trials, with improved reporting of trials. Over the last 3 to 4 years several new trials and systematic reviews have been published [12-15]. To date many trials are heterogeneous and results inconsistent. There are several possible explanations for the heterogeneity of trials included in the systematic reviews. Several reviews included a trial addressing a different research question to other trials [16]. This trial also reported a very high pregnancy rate in the control group. The heterogeneity may also be attributed to the combining of data from different control groups, for example groups including IVF alone and no acupuncture, the use of needles inserted into sham points, and the use of non-invasive placebo needles. There was also clinical heterogeneity in the treatment protocols used between studies, with variation in the acupuncture points used, timing of treatment, and frequency of acupuncture. There remains insufficient evidence to determine whether acupuncture can enhance live birth rates when used as an adjunct to IVF treatment.

Our study aimed to reduce the methodological and clinical heterogeneity observed in previous designs. We designed a multicentered, randomized controlled trial (RCT) that compares acupuncture with an acupuncture control, and a non-randomized observational usual care group. We undertook a Delphi study to achieve consensus on the design of the optimal acupuncture treatment protocol to be used in this research. The primary study objective is to determine the clinical effectiveness of acupuncture with improving the proportion of women undergoing IVF having live births, the key outcome in trials of reproductive medicine. Secondary study objectives include: methods to determine the cost-effectiveness of IVF with acupuncture; and a qualitative study to examine the personal and social context of acupuncture in IVF patients, examining the reasons why the acupuncture may or may not have worked, and identify other effects of acupuncture.

## **Methods/design**

We will conduct a parallel randomized controlled trial of acupuncture compared with a placebo control using a non-invasive placebo needle. A non-randomized group will receive standard care to allow comparisons with the baseline pregnancy rate. The rationale for a non-randomized arm includes the need to provide a baseline pregnancy rate, and acknowledgement from our previous research experience that women would not accept randomization to this group [17]. We acknowledge this group of women may have different outcomes, and incorporating a usual care group to provide baseline pregnancy rate is problematic. The analysis of the usual care only group will be treated as an observational study with known confounding factors adjusted for in the analysis. The study will recruit participants from IVF centers in Australia (IVF Australia, Assisted Conception and Fertility South Australia, Flinders Reproductive) and one center in New Zealand (Fertility Plus).

### **Eligibility criteria**

We will recruit women aged <43 years, undergoing a fresh IVF or intracytoplasmic sperm injection (ICSI) cycle, and restricted to women with the potential for a lower live birth rate defined as two or more previous unsuccessful ETs (fresh or frozen), and unsuccessful clinical pregnancies of quality embryos deemed by the embryologist to have been suitable for freezing by standard criteria. The rationale for our inclusion criteria is based on a possibility that where the baseline pregnancy rates are high for some IVF settings, the added value of acupuncture maybe reduced. We have identified a group of women were the pregnancy rates are lower, and for whom adding acupuncture may improve their clinical outcomes. The characteristics of age <43 years, undergoing a fresh IVF or intracytoplasmic sperm injection (ICSI) cycle, reflect the characteristics of majority of women undergoing fresh IVF cycle in Australia and New Zealand. IVF units outside of Australia and New Zealand may have lower age restrictions.

Women will be excluded if they are undergoing a frozen ET, have been previously randomized to the study, planning pre-implantation genetic diagnosis, or receiving donor eggs, or are currently having acupuncture.

### **Randomization and blinding**

Women will be allocated to a study group by a research nurse phoning the randomization service, prepared and based at the National Health and Medical Research Council Clinical Trials Centre, Sydney, Australia. The randomization sequence is computer generated and concealed via central allocation. There will be stratification by the number of ET cycles (2 to 6 and 6+), woman’s age (<38 years, and 38 to 42 years), and collaborating center. Randomization will be into two study groups, acupuncture and a placebo control using the non-invasive Park needle [18]. Study participants, care providers, the outcome assessors, and the analyst will be blind to study group allocation.

### **Interventions**

Following randomization women in the intervention groups will see the study acupuncturist based at the IVF centers, or at close proximity to the IVF centers. The acupuncturists are registered with national professional associations, and with a minimum of 2 years clinical experience. Training will be provided to all study acupuncturists on the treatment protocol, practitioner intent or treatment aspirations, recording of treatments administered, the acupuncturists’ duty and an explanation of proposed future monitoring. The treatment protocol was based on two rounds of a Delphi process, with consensus achieved on a treatment protocol for the study. Nineteen acupuncturists participated in this process, with practitioners from Australia (6), China (2), Denmark (1), Sweden (1), the United States (4), and the United Kingdom (1). All women will undergo a traditional Chinese medicine (TCM) diagnosis. The initial diagnosis and treatment will take 60 to 90 minutes undertaken on days 6 to 8 of the stimulated IVF cycle. Two treatments will be administered immediately before and after ET.

Group 1: Acupuncture treatment based on the traditional Chinese medicine (TCM) style of acupuncture. For women randomized to receive acupuncture the initial treatment includes core points Guilai ST-29, Guanyuan Ren-4, Qihai Ren-6, Sanyinjiao SP-6, Xuehai SP-10, plus up to five additional points based on a TCM pattern differentiation. Two subsequent treatments will be administered on the day of ET pre and post transfer. Points administered on the day of ET include Diji SP-8, Xuehai SP-10, Taichong LR-3, Guanyuan Ren-4, one point from Shenmen HT-7, Neiguan PC-6, or YinTang, Baihui DU-20, Taixi KD-3, Zusanli ST-36, Sanyinjiao SP-6, and auricular points Shenmen and Zhigong. Manual acupuncture will be performed with needles inserted using the Park device [18], a supporting tube that facilitates maintenance of blinding for the participant. Needles will be inserted to a depth of not greater than 2 cm and retained for 25 min. The insertion of the acupuncture needle into an acupuncture point typically generates a range of sensations called ‘de qi’, this sensation will be maintained during the initial treatment on days 6 to 8, and during the pre-embryo treatment only. Points will be inserted bilaterally except for acupuncture points located on the Ren and Du points and YinTang point.

Group 2: Placebo control. This group will receive placement of non-invasive Park sham needles [18]. The Park needle has been shown to be an effective device for blinding in RCT [18]. These needles have a retractable needle shaft, a blunt tip, and skin penetration does not occur, and the needles have a supporting device. The acupuncturist will hold the ‘needle’ in place with one hand, while moving the handle of the needle with the other hand, so the shaft disappears into the handle. The needle is inserted through Park supporting device. The location of sham non-acupuncture points are away from real points and are described in relation to anatomical landmarks and relationship to acupuncture channels. Thirteen sham points are described: three on the abdomen, four on the arm, two on the leg, one on the back, one on the forehead, one on the foot, and one on the ear. The duration of needling and treatment session is the same as for the acupuncture group.

Study group 3: Usual care only. For women who decline to be randomized and were not planning to have acupuncture, they will be invited to participate in a non-randomized group receiving standard care only group, and provide consent to participate in all data collection.

### **Outcome measures**

**Figure 1:**
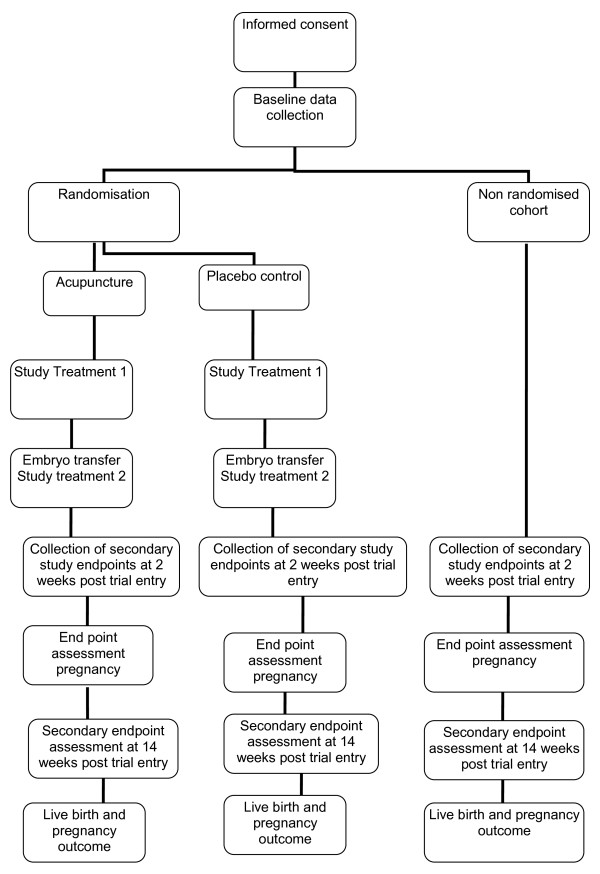
Study design.

Our primary study endpoint will be the proportion of women reporting a live birth defined as the delivery of one or more living infants, >20 weeks gestation or 400 g or more birth weight. Our secondary endpoints will be the proportion of women reporting a clinical pregnancy defined as demonstration of fetal heart activity on ultrasound scan, measured at 7 to 8 weeks, miscarriage defined as a non-viable pregnancy prior to 12 weeks, quality of life using the MOS Short Form 36 (SF36) [19], anxiety using the Stait Trait Anxiety Inventory [20] and infertility self-efficacy measured by the Infertility Self-Efficacy Scale [21] at trial entry, 2 and 14 weeks from trial entry. We will also collect data on expectations, and the therapeutic alliance. Clinical data will be collected from the IVF center database. Other outcomes will be collected by postal questionnaires. Data on the safety of acupuncture and any adverse events will be collected from practitioner treatment notes (Figure 1**)**

### **Sample size**

Our pilot data [17] were suggestive of a larger treatment difference; however we have chosen a more conservative 7% estimate of the clinical effect from acupuncture compared with the placebo control group. The proportion of women with a live birth for women with multiple cycle failures in the placebo control group in our previous study was 12.1% [17]. To detect a 7% increase in the proportion of women that report a live birth between the treatment and placebo control, with 80% power at the 5% significance level will require 449 women per group. We have allowed for a loss of 30% due to cancelled cycles, or no ET. A total sample size of 1,168 women is required.

We expect a larger treatment effect (10%) when comparing acupuncture and standard care. To detect a 10% increase in the proportion of women that report a live birth between the treatment and standard care, with 80% power at the 5% significance level will require 193 women in the non-randomized group. Allowing for a 30% loss, due to cancelled cycles, or no ET, 251 women in the non-randomized group are required.

### **Data analysis**

Data will be analyzed by a statistician based at UWS blind to study group. Analyses of the endpoints will undertake an ‘intention to treat’ approach and compare differences in the primary and secondary endpoints between groups. The primary analysis will compare the proportions of patients with live births. Logistic regression will be used to identify baseline variables which are associated with outcomes. Linear models will be used to test for between group differences in the continuous outcome measures (for example, health status).

Subgroup analyses will be stated *a priori*, for example influence of stimulation doses, long- or short-acting stimulation cycle. Levels of significance will be reported at *P* < 0.05.

### **Qualitative study**

A qualitative study using in-depth interviews is nested within the RCT. The purpose of the interviews is: to investigate how women perceive the study intervention and how they think about their use and experience of it, and its effects; and to provide evidence of the process by which the outcomes of the RCT may have been achieved. A sample of 50 women will be recruited through theoretical and purposive sampling to ensure equal numbers of participants from both the intervention and placebo control groups, and a socially diverse sample of women across demographic background, treatment outcome and coping outcomes (as determined by the self-efficacy scores). It is anticipated that 50 interviews will lead to data saturation. The interview will cover topics such as the women’s experience of IVF treatment and its impact on personal wellbeing and social and family relationships, and women’s perception of the impact of acupuncture. Interviews will examine their experience during the period following ET and the pregnancy test. Their beliefs about the impact of acupuncture on their bodies and health, and its potential impact on the reproductive outcome of their treatment will be explored in conversation. Analysis of qualitative interview data will be iterative and the interview schedule will be modified to include emerging themes.

### **Economic analysis**

An economic analysis will be conducted alongside the RCT. The primary measure of outcome for the economic analysis will be the cost per live birth. Resource use collected within the study will include costs associated with the provision of the proposed acupuncture intervention plus the direct costs of IVF based on expected average clinic fees, the frequency and duration of GP visits, the frequency and duration of antenatal visits and inpatient admissions, for example miscarriage loss or antenatal complications. Mean costs and effectiveness between the intervention and control groups will be compared and incremental cost-effectiveness ratios (ICERs) presented in terms of the cost per unit improvement in clinical outcome as measured by the live birth rate. An assessment of the sensitivity of the results obtained to variation in measured resource use, effectiveness and/or unit costs will be undertaken using appropriate one-way and multi-way sensitivity analysis [22]. Data will be collected from Medicare, a national database of health utilization, activity and resource use, and completion of data forms by women reporting health utilization not captured by Medicare.

### **Ethics**

Approval to conduct this study has been obtained from the following research and ethics committees: University of Western Sydney (New South Wales), Greenslopes Private Hospital (Brisbane, Queensland), IVF Australia (Sydney, New South Wales), St Andrews Hospital (Adelaide, South Australia), and the Southern Adelaide Clinical Human Research Ethics Committee (South Australia).

## **Discussion**

IVF is a costly treatment option for women, their partners and the public, and there is a significant personal cost to couples undergoing repeated IVF cycles. Therefore new therapies that improve reproductive and health outcomes are highly desirable. Acupuncture is an emerging therapy used as an adjunct to IVF, and it is important to healthcare consumers and healthcare providers that the evidence base for this modality be established.

This research will add significant new knowledge to defining the role of certain acupuncture protocols applied on the day of ET and one other day of the IVF from a clinical and cost-effectiveness perspective. The study design utilizing mixed methods will also generate a different form of new knowledge that will allow the social and personal experience of acupuncture to be explained and their relationship to acupuncture determined. The research will add significantly to the clinical evidence base to allow conclusions to be made on the role of acupuncture to improve reproductive outcomes.

### **Trial Status**

This trial is currently recruiting participants.

## **Competing interests**

The authors declare that they have no competing interests.

## **Authors’ contributions**

CAS, SdL, MC, JR, RN, NJ, GS, JL, and CB all contributed to the development of the study protocol. CAS prepared the initial draft of the manuscript, and all author critically reviewed the content. All authors approved the final version.
