# Erratum to Acupuncture to improve live birth rates for women undergoing *in vitro* fertilization: a protocol for a randomized controlled trial

**DOI:** 10.1186/s13063-017-1986-4

**Published:** 2017-06-01

**Authors:** Caroline A. Smith, Sheryl de Lacey, Michael Chapman, Julie Ratcliffe, Robert J. Norman, Neil Johnson, Gavin Sacks, Jane Lyttleton, Clare Boothroyd

**Affiliations:** 1 Centre for Complementary Medicine Research, University of Western Sydney, Locked Bag, 1797, Penrith South, DC, NSW 2751 Australia; 2 School of Nursing & Midwifery, Flinders University, GPO Box 2100, Adelaide, South Australia 5001 Australia; 3 School of Women’s and Children’s Health, University of New South Wales, St George Hospital, IVF Australia Southern Sydney, South Street, Kogarah, NSW 2217 Australia; 4 Flinders Clinical Effectiveness, Flinders University, Bedford Park, South Australia 5001 Australia; 5 Robinson Institute, University of Adelaide, Norwich House, 55 King William Road, North Adelaide, South Australia 5006 Australia; 6 Fertility Plus, Auckland District Health Board; Repromed Auckland, University of Auckland, Auckland, New Zealand; 7 School of Women’s and Children’s Health, University of New South Wales, St George Hospital, South Street, Kogarah, New South Wales 2217 Australia; 8 Paddington Medical Centre, 198 Oxford St, Paddington, New South Wales 2021 Australia; 9 Assisted Conception Australia, Greenslopes Private Hospital, Suite 9A, Administration Building, Brisbane, QLD 4120 Australia

## Erratum

*After publication of our article* [1] *we realised that our Competing Interests statement should have read as follows:*

*Jane Lyttleton is the Clinical Director of The Acupuncture Pregnancy Clinic*

*Caroline Smith has had an association with The Acupuncture Pregnancy Clinic. She states that she has not received any financial compensation for this relationship at any time.*
